# Non‐small cell lung cancer with *EGFR* (L858R and E709X) and CNNB1 mutations responded to afatinib

**DOI:** 10.1111/1759-7714.14775

**Published:** 2022-12-15

**Authors:** Michihiro Kunishige, Seiya Ichihara, Naoki Kadota, Yoshio Okano, Hisanori Machida, Nobuo Hatakeyama, Keishi Naruse, Tsutomu Shinohara, Eiji Takeuchi

**Affiliations:** ^1^ Department of Respiratory Medicine National Hospital Organization Kochi Hospital Kochi city Kochi Japan; ^2^ Department of Pathology National Hospital Organization Kochi Hospital Kochi city Kochi Japan; ^3^ Department of Community Medicine for Respirology, Graduate School of Biomedical Sciences Tokushima University Tokushima Japan; ^4^ Department of Clinical Investigation National Hospital Organization Kochi Hospital Kochi city Kochi Japan

**Keywords:** afatinib, CTNNB1 mutation, E709X, L858R, non–small cell lung cancer

## Abstract

Lung cancer with complex epidermal growth factor receptor (EGFR) and CTNNB1 comutations is rare, and the efficacy of tyrosine kinase inhibitors (TKIs) is generally poor. Here, we encountered a lung cancer patient with complex EGFR (L858R and E709X) and CTNNB1 comutations who successfully responded to afatinib. A 78‐year‐old woman visited our hospital with a cough and bloody sputum that had worsened over the past year. She had multiple mass shadows in both lungs and nodular shadows in the bronchi. The patient was diagnosed with lung adenocarcinoma cT4N3M1c stage IVB. A genetic analysis of the primary tumor using the Oncomine Dx target test multi‐CDx system revealed positivity for *EGFR* (L858R and E709X) and CTNNB1 mutations. The expression of programmed death ligand 1 (22C3 clones) in tumor cells was negative by immunostaining. The patient was treated with afatinib as first‐line therapy and achieved clinical improvement and a partial response and is continuing treatment 1 year later. Case reports of lung cancer patients with EGFR/CTNNB1 comutations are rare, and TKIs are not considered to be effective. We herein present the first case report of lung cancer with the co‐occurrence of uncommon and complex *EGFR* (L858R and E709X) and CTNNB1 mutations that was successfully treated with afatinib.

## Graphical abstract

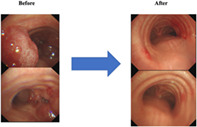

## INTRODUCTION

The use of epidermal growth factor receptor (EGFR) tyrosine kinase inhibitors (TKIs) to treat *EGFR* mutation‐positive non‐small cell lung cancer (NSCLC) has markedly improved its prognosis.^1^, ^2^, ^3^, ^4^ However, previous studies have demonstrated that disease progression was earlier and overall survival was shorter in patients with uncommon *EGFR* mutations than in those with common *EGFR* mutations.^5^, ^6^ Afatinib has previously been shown to be effective against NSCLC tumors with some uncommon *EGFR* mutations.^7^, ^8^ CTNNB1 mutations have previously been detected in 5.3% of lung adenocarcinomas and 2% of NSCLC.^9^, ^10^ They have been identified as a critical resistance mechanism to EGFR‐TKIs.^11^, ^12^ However, there have been no case reports of lung cancer with uncommon and complex *EGFR* (L858R and E709X) and CTNNB1 comutations. We herein report the first case report of lung cancer with the co‐occurrence of uncommon and complex *EGFR* (L858R and E709X) and CTNNB1 mutations that was successfully treated with afatinib.

## CASE REPORT

**FIGURE 1:**
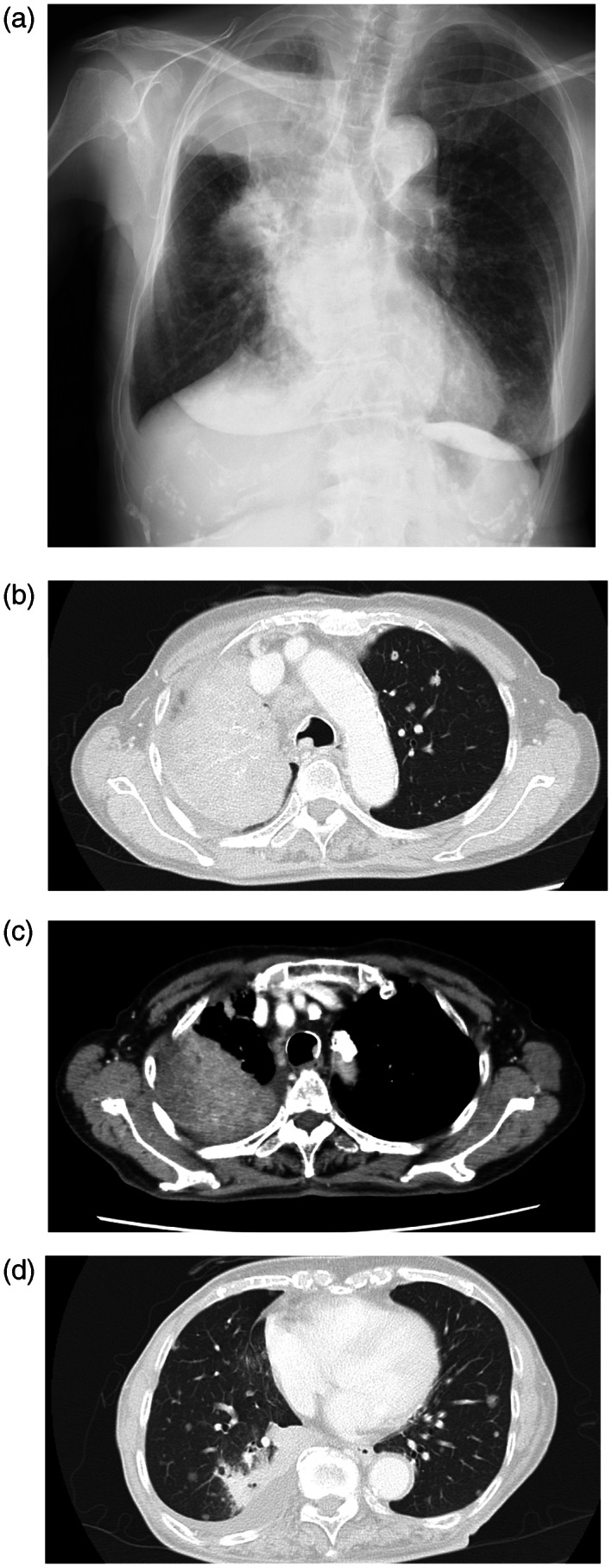
(a) Chest X‐ray and (b, c, d) contrast‐enhanced computed tomography (CT) at first admission. Mass shadows in the right upper, middle, and lower lobes, multiple nodular shadows in both lungs, and intramural nodules in the trachea and right bronchus. Right pleural effusion was noted.

**FIGURE 2:**
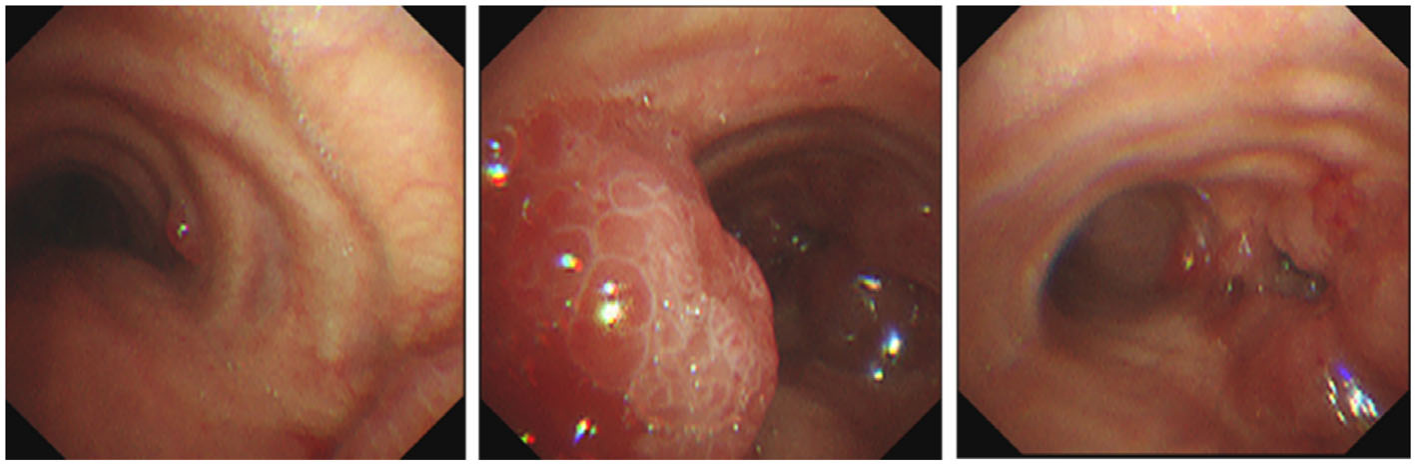
Bronchoscopy with standard observation revealed multiple tumors in the trachea and a tumor which obstructed the right main bronchus.

**FIGURE 3:**
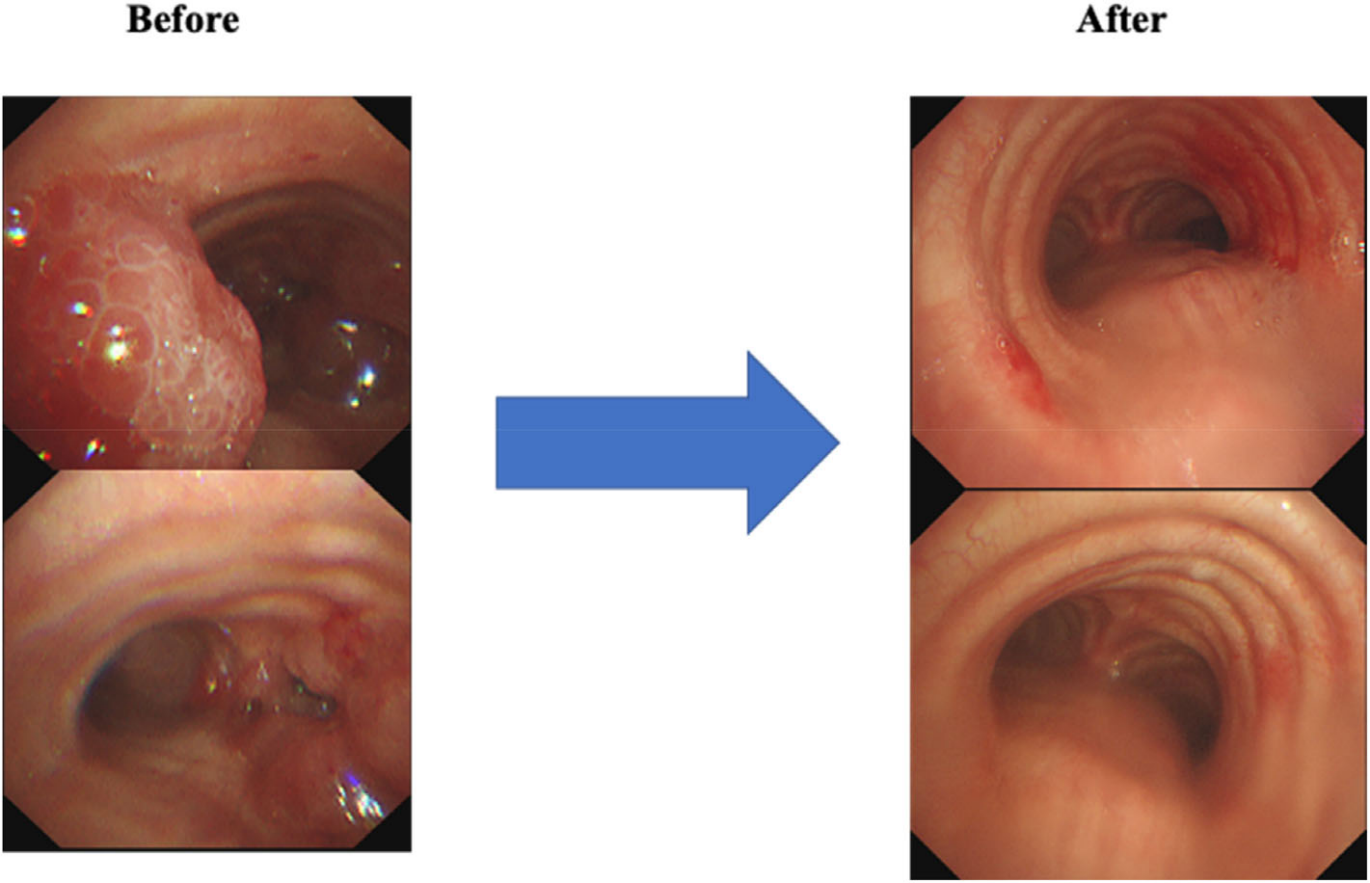
Bronchoscopy findings before and 7 months after treatment.

**FIGURE 4:**
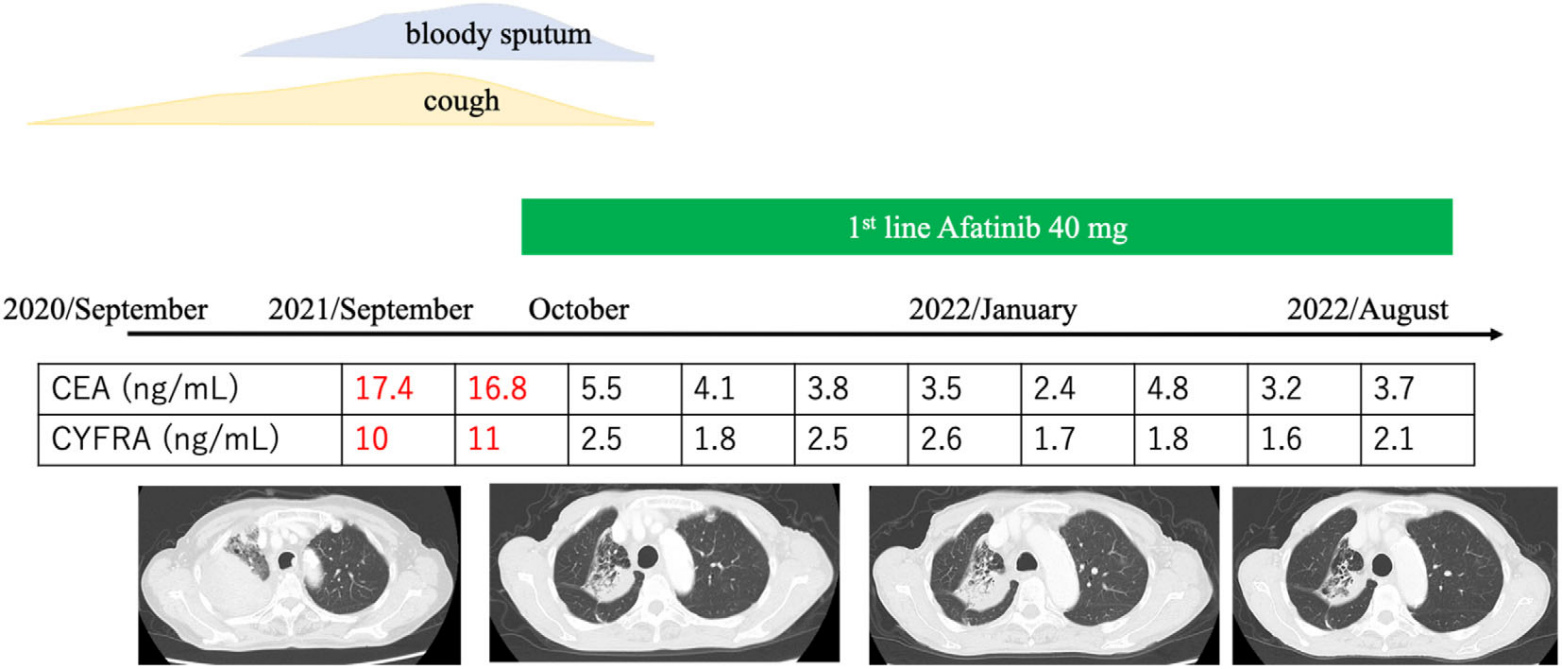
The clinical course of the patient.

A 78‐year‐old woman was referred to our hospital with a cough and bloody sputum. She was a nonsmoker with a family history of lung cancer. She also had a history of hypertension. Her Eastern Cooperative Oncology Group performance status was one. The results of blood tests were as follows: white blood cell count 11 600/μl, red blood cell count 403 × 10^4^/μl, platelet count 24.0 × 10^4^/μl, carcinoembryonic antigen 17.4 ng/ml, and cytokeratin 19 fragments 10.0 ng/ml. Chest X‐ray (Figure FIGURE 1a) and contrast‐enhanced computed tomography (CT) (Figure FIGURE 1b‐d) showed mass shadows in the right upper, middle, and lower lobes, multiple nodular shadows in both lungs, and intramural nodules in the trachea and right bronchus. Bronchoscopy with standard observations revealed multiple tumors in the trachea, and a tumor which obstructed the right main bronchus (Figure FIGURE 2). Transbronchial tumor biopsies showed adenocarcinoma. Contrast‐enhanced computed tomography showed metastases in the lung, pleura, and liver. The patient was diagnosed with right upper lobe adenocarcinoma cT4N3M1c stage IVB. A genetic analysis of the primary tumor using the Oncomine Dx target test multi‐CDx aystem revealed positivity for *EGFR* (L858R and E709X) and CTNNB1 mutations. The allele frequencies of L858R, E709X, and CTNNB1 were 14.8, 14.7, and 13.7%, respectively. The expression of programmed death ligand 1 (22C3 clones) in tumor cells was negative by immunostaining. Based on these findings, the patient was treated with afatinib (40 mg/day) as first‐line therapy. She achieved clinical improvement and a partial response. The only side effects that developed were grade 1 diarrhea and skin rash, and, thus the patient continued treatment. Seven months later, bronchoscopy showed that the tumor in the trachea had disappeared (Figure 3). One year later, other tumors had shrunk and tumor markers had normalized (Figure FIGURE 4).

## DISCUSSION

We encountered a lung cancer patient with uncommon and complex *EGFR* (L858R and E709X) and CTNNB1 comutations. To the best of our knowledge, this is the first case report of lung cancer with the co‐occurrence of uncommon and complex *EGFR* (L858R and E709X) and CTNNB1 mutations. This patient successfully responded to afatinib.

EGFR‐TKIs for *EGFR* mutation‐positive NSCLC have markedly improved its prognosis.^1^, ^2^, ^3^, ^4^ Progression‐free survival after EGFR‐TKI therapy has previously been reported to be significantly longer in patients with a mutant allele frequency of L858R (MAFLR) of >9% than in those with a MAFLR of ≤9%.^13^ However, previous studies demonstrated that disease progression was earlier and overall survival was shorter in patients with uncommon *EGFR* mutations than in those with common EGFR mutations.^5^, ^6^ Afatinib was found to be effective against NSCLC tumors with some uncommon *EGFR* mutations.^7^, ^8^ It has also been recommended for complex mutations involving E709X, S768I, or G719X.^14^ The present case with uncommon and complex EGFR (L858R and E709X) and CTNNB1 comutations responded to afatinib and has continued treatment for 1 year.

Beta‐catenin, encoded by the CTNNB1 gene, is essential for establishing and maintaining the epithelial layer and is a key downstream component of the canonical Wnt signaling pathway.^15^ CTNNB1 mutations are oncogenic in several cancers.^15^ CTNNB1 mutations in the Wnt/β‐catenin pathway and RAS, RAF, and upstream *EGFR* mutations in the RAS–ERK pathway have been shown to play essential roles in tumorigenesis.^16^ Among 564 patients with lung adenocarcinoma, 30 (5.3%) harbored CTNNB1 mutations.^9^ Another study detected CTNNB1 mutations in 11 (2%) out of 546 NSCLC patients, commonly in conjunction with *EGFR* mutations.^10^ A previous study reported that females and nonsmokers with lung cancer were more likely to harbor CTNNB1 mutations.^9^ CTNNB1 mutations are an important factor in EGFR‐TKI resistance.^11^, ^12^ Furthermore, they have been shown to contribute to the development of a noninflamed tumor microenvironment.^17^, ^18^ Recurrence‐free survival was found to be significantly shorter in patients with CTMNNB1/EGFR comutations than in those with a single *EGFR* mutation.^19^ In lung adenocarcinomas, CTNNB1 expression is associated with shorter survival.^20^ Therefore, our patient needs to be carefully observed.

In conclusion, we present a lung cancer patient with uncommon and complex *EGFR* (L858R and E709X) and CTNNB1 comutations. The patient successfully responded to afatinib and has continued treatment for 1 year. CTNNB1 mutations have been identified as a crucial resistance mechanism to EGFR‐TKIs, and, thus, careful observations are warranted.

## AUTHOR CONTRIBUTION

MK drafted the original manuscript. ET reviewed the manuscript draft and revised it critically on intellectual content. SI, NK, YO, HM, NH, KN and TS reviewed the manuscript draft. All authors approved the final version of the manuscript to be published.

## CONFLICT OF INTEREST

The authors declare no conflicts of interest.
